# First Brazilian recommendation on physiotherapy with sensory motor stimulation in newborns and infants in the intensive care unit

**DOI:** 10.5935/0103-507X.20210002

**Published:** 2021

**Authors:** Cíntia Johnston, Mônica Sanchez Stopiglia, Simone Nascimento Santos Ribeiro, Cristiane Sousa Nascimento Baez, Silvana Alves Pereira

**Affiliations:** 1 Faculdade de Medicina, Universidade de São Paulo - São Paulo (SP), Brazil.; 2 Hospital da Mulher Prof. Dr. José Aristodemo Pinotti, Universidade Estadual de Campinas - São Paulo (SP), Brazil.; 3 Faculdade de Ciências Médicas de Minas Gerais - Belo Horizonte (MG), Brazil.; 4 Instituto Federal de Educação, Ciência e Tecnologia do Rio de Janeiro - Rio de Janeiro (RJ), Brazil.; 5 Universidade Federal do Rio Grande do Norte - Natal (RN), Brazil.

**Keywords:** Infant, Infant, newborn, Sensory motor stimulation, Neuropsychomotor development, Child development, Psychomotor performance, Intensive care units, neonatal, Lactente, Recém-nascido, Estimulação sensório-motora, Desenvolvimento neuropsicomotor, Desenvolvimento infantil, Desempenho psicomotor, Unidades de terapia intensiva, neonatal

## Abstract

**Objective:**

To present guidelines on sensory motor stimulation for newborns and infants in the intensive care unit.

**Methods:**

We employed a mixed methods design with a systematic review of the literature and recommendations based on scientific evidence and the opinions of physiotherapists with neonatal expertise. The research included studies published between 2010 and 2018 in the MEDLINE® and Cochrane databases that included newborns (preterm and term) and infants (between 28 days and 6 months of age) hospitalized in the intensive care unit and submitted to sensory motor stimulation methods. The studies found were classified according to the GRADE score by five physiotherapists in different regions of Brazil and presented at eight Scientific Congresses held to discuss the clinical practice guidelines.

**Results:**

We included 89 articles to construct the clinical practice guidelines. Auditory, gustatory and skin-to-skin stimulation stand out for enhancing vital signs, and tactile-kinesthetic massage and multisensory stimulation stand out for improving weight or sucking.

**Conclusion:**

Although all modalities have good ratings for pain or stress control, it is recommended that sensory motor stimulation procedures be tailored to the infant’s specific needs and that interventions and be carried out by expert professionals.

## Abstract (pt)

**Objetivo:**

Apresentar as diretrizes de estimulação sensório-motora para recém-nascidos e lactentes em unidade de terapia intensiva.,

**Métodos:**

Trata-se de um método de delineamento misto com revisão sistemática da literatura e recomendações com base na evidência científica e opiniões de fisioterapeutas especialistas em fisioterapia neonatal de estudos publicados entre 2010 e 2018 nas bases de dados MEDLINE® e Cochrane, que incluiu recém-nascidos (pré-termo e a termo) e lactentes (entre 28 dias e 6 meses de idade) admitidos à unidade de terapia intensiva e submetidos a métodos de estimulação sensório-motora. Os estudos encontrados foram classificados segundo o escore GRADE por cinco fisioterapeutas em diferentes regiões do país e apresentados em oito congressos científicos para discussão das diretrizes de práticas clínicas.,

**Resultados:**

Foram incluídos 89 artigos para construir as diretrizes de práticas clínicas. Estimulação auditiva, gustatória e contato pele a pele se destacaram por melhorar os sinais vitais, e a massagem terapêutica, assim como a estimulação multimodal tátil-cinestésica por melhorar o peso ou a sucção.,

**Conclusão:**

Embora todas a modalidades tenham boas avaliações para controle da dor ou do estresse, é recomendado que os procedimentos de estimulação sensório-motora sejam adaptados às necessidades específicas da criança, e as intervenções sejam realizadas por profissionais experientes.

## INTRODUCTION

Sensory motor stimulation (SMS) for newborns (preterm or term) and infants in the intensive care unit (ICU) is an early intervention that includes a series of strategies aimed at optimizing neuropsychomotor development (NPMD) by promoting sensory stimuli based on the level of functional development, gestational age (GA) at birth, and weight of this population.^(1)^

The primary aim of SMS is to organize human body systems. i.e., tactile, kinesthetic, vestibular, olfactory, taste, auditory, visual and/or a combination of these.^(1)^ In the ICU, newborns and infants are often in moderately to highly complex clinical situations that may lead to unstable neurological, hemodynamic and cardiorespiratory systems, requiring technical and scientific knowledge when conducting overall assessments of SMS candidates.^(2,3)^

Despite technological advances and multiprofessional efforts, extremely premature (GA < 28 weeks) and extremely low weight (< 1,000g) newborns remain at high risk of death and functional disability (short, mid and long term). Approximately 20% to 50% of survivors are at risk of morbidity, including changes in weight-height growth and NPMD.^(2)^

Sensory motor stimulation facilitates typical NPMD and prevents or minimizes the harmful effects of the ICU environment and interventions on weight-height growth. As such, it can be applied to treat NPMD changes resulting from prematurity, diseases and/or alterations/complications in the prenatal, perinatal or intranatal period and postdelivery.^(4-6)^

The aim of the present study is to present clinical practice guidelines on SMS for newborns and infants in the ICU.

## METHODS

### Study design

We employed a mixed method design and the following four stages to create this document.

**Stage 1 -** subject approval for the creation of this document and classification of SMS into the following:

**Recommendation**: the main findings are based on at least one clinical trial, considering scientific evidence on the benefits *versus* risks to newborns and infants hospitalized in the ICU, viability of comparisons with other intervention options, and confirmation of the reliability of the evidence presented to support the use or rejection of SMS in the clinical practice of physiotherapists.

**Guiding question:** the PICO domains are considered: **P**, Patient (newborn or infant); **I**, Intervention (any SMS intervention); **C**, Comparison (cross-sectional or prospective longitudinal comparison with itself; with the control, with no intervention or placebo; or with another SMS intervention) and **O**- Outcome (studies including weight-height outcomes and their indices; improved sleep quality; reduced pain; increase in any NPMD domain; other NPMD-related outcomes - example: arm circumference, bone growth - and weight-height growth).

**Stage 2** - a systematic search was conducted of the Medline and Cochrane databases for studies on SMS published between 2010 and 2018. The keywords used included controlled indexers contained in Health Sciences Descriptors (DeCS, available at http://decs.bvs.br/P/decsweb2014.htm) and/or in Medical Subject Headings (MeSH, available at http://www.ncbi.nlm.nih.gov/entrez/query.fcgi?db=mesh); a number of free terms related to each SMS modality were also used (see below each subitem of the Recommendations in “descriptors”). The search terms were combined using the Boolean operators “OR” and “AND” and their corresponding Portuguese words. Five specialists conducted the systematic search and assessed the studies independently according to each SMS modality. Disagreements were resolved by common consent of all those present during the discussions and/or via Skype. The specialists were subdivided into pairs to write a report on the SMS interventions. All specialists were physiotherapists with neonatal expertise (experience ≥ 12 years) in SMS for newborns and infants (up to six months of age) in the ICU.

**Stage 3** - the partial data were presented to the public at different pediatric and neonatology congresses, where participants could give their opinions and offer suggestions and comments. The five specialists analyzed the suggestions and comments provided by the public at the aforementioned events and made pertinent changes to the document.

**Figure 1:**
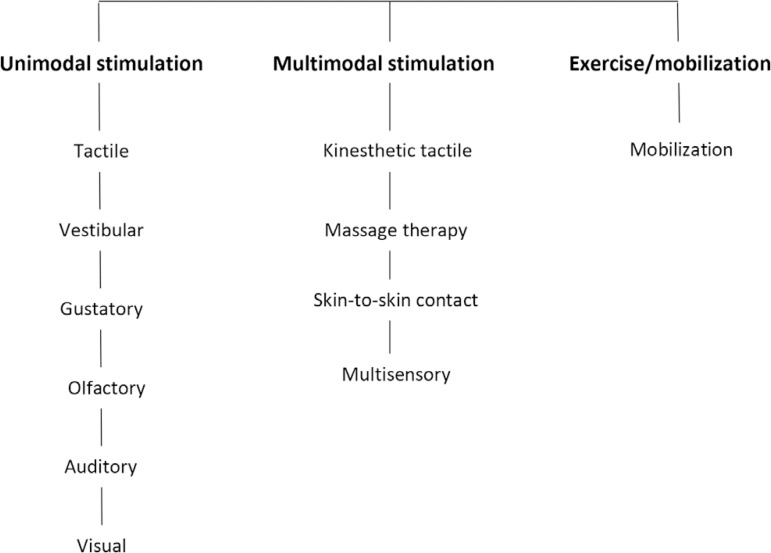
Sensory motor stimulation modality recommendations for newborns and infants hospitalized in neonatal intensive care.

**Stage 4** - creation and writing of the document, in line with the three SMS modalities (unimodal and multimodal stimulation and exercises/mobilizations) and the types of interventions found in the literature (Figure 1).

### Inclusion and exclusion criteria

Clinical trials that met the following criteria were included: (1) clinical study, controlled or not, comparative or not, randomized or not, or crossover; (2) the study included some type of SMS intervention; (3) study population consisting of newborns and/or infants and (4) neonatal ICU as the study site. Duplicate articles and review studies, case reports, editorials and letters to the editor were excluded. When deemed relevant, these were included in the introduction and/or comments of the document.

### Quality assessment

The studies found were classified according to the modified Grading of Recommendations Assessment, Development and Evaluation (GRADE) score.^(7)^ GRADE is the evidence rating system endorsed by the World Health Organization.^(7)^ It rates the quality of evidence on a 4-point scale (high, moderate, low and very low). Randomized trials start at a score of 4/4 (high) and can be downgraded based on methodological flaws. If no published literature was available, expert physiotherapists’ opinions were used. The GRADE ratings are shown in table 1, with clinical indicators presented by + signs, indicating the degree of scientific certainty: +, the recommendation was very weak; ++, weak; +++, moderate and ++++, strong.

**Table 1:** Classification of clinical indicators, scientific certainty and recommendations for sensory motor stimulation

Clinical indicators	Tactile	Auditory	Olfactory	Gustatory	Tactile-kinesthetic*	Massage*	Skin-to-skin*	Multisensory*	Mobilizations*
Reduce pain/stress or improve behavioral organization	+++	+++	+++	++++	+++	++++	++++	++++	
Improve vital physiological events (regulate RR; HR, SapO_2_, temperature and reduce apnea episodes)	++	++++	+++				++++		
Improve sleep-wake cycles	++	+			++	+			
Accelerate brain maturation					+	+		+++	
Improve weight or sucking or promote faster progression to total oral feeding		++			+++	+++	++++	+++	
Improve bone mass or muscle strength or muscle tone maturation					+++			+++	+++
Decrease length of hospitalization or reduce the number of morbidities		++			+++	+++	++++		

RR - respiratory rate; HR - heart rate; SpO_2_ - blood oxygen saturation. Degree of scientific certainty: ++++, strong; +++, moderate; ++, weak; +, very weak.

* Multimodal stimulation = sensory motor stimulation interventions that combine two or more types of sensory stimuli.

## RESULTS

A total of 89 articles were included. The partial data were presented at different pediatric and neonatology congresses: The Brazilian Congress of Intensive Therapy (*Congresso Brasileiro de Medicina Intensiva* - CBMI-AMIB), Florianópolis (SC), 2014; the International Symposium of Cardiorespiratory Physiotherapy (*Simpósio Internacional de Fisioterapia Cardiorrespiratória*), Salvador (BA), 2014; CBMI-AMIB, Goiânia (GO), 2014; CBMI-AMIB Costa do Sauípe (BA), 2015; CBMI-AMIB Porto Alegre (RS), 2016; CBMI-AMIB Natal (RN), 2017; Pan-American Congress of Intensive Therapy Rio de Janeiro (RJ), 2017; CBMI-AMIB São Paulo (SP), 2018 and CBMI-AMIB Fortaleza (CE), 2018, with the participation of approximately 600 physiotherapists in the area of neonatal and pediatric intensive therapy from different regions of Brazil.

The clinical indicators classified by GRADE are shown in table 1. The tables 1S to 9S in the appendix 1 shows a summary of the data for the studies included.

## DISCUSSION

### Unimodal stimulation

Unimodal stimulation includes SMS interventions that provide only one type of sensory stimulation to newborns or infants, in line with the physiological development hierarchy of sensory subsystems, such as tactile→vestibular→taste→olfactory→auditory→visual.^(6)^

#### Tactile stimulation

**Recommendation:** tactile stimulation is recommended to reduce stress, assessed by urine cortisol level, and applied using the gentle human touch (GHT) intervention;^(8)^ reduce pain intensity, as assessed by the Neonatal Infant Pain Scale (NIPS) and changes in heart rate (HR) and respiratory rate (RR) associated with pain stimuli, using the therapeutic touch (TT) intervention;^(9)^ and improve sleep state, as assessed by the Anderson Behavioral State Scale (ABSS) after the GHT intervention and the Yakson protocol.^(10)^ The clinical indicators classified by GRADE are shown in table 1.

Vestibular stimulation

**Recommendation:** some functional positioning methods, which can also be used for vestibular stimulation (for example, hammocks, frequently used in the ICU in Brazil), did not exhibit the degree of scientific evidence required for inclusion in unimodal stimulation and were therefore included in multimodal SMS.

#### Auditory stimulation

**Recommendation:** auditory stimulation is recommended to increase peripheral capillary oxygen saturation (SpO_2_) and to reduce HR through exposure to male-sung lullabies;^(11)^ increase SpO_2_ through exposure to a Brahms’ lullaby or one sung/recorded by the mother;^(12)^ decrease physiological (HR) and behavioral responses (sleep-wake state and facial expressions of pain) during and after pain stimuli;^(13,14)^ decrease resting energy expenditure through exposure to Mozart’s music (Mozart effect);^(15)^ lower HR and RR through exposure to lullabies and reduce HR during exposure to Mozart’s music;^(16)^ lower HR and RR using three types of interventions (lullabies, heartbeat-like sounds and sounds resembling breathing), better sucking behavior with heartbeat-like sounds and a rise in caloric intake and improved feeding behavior (sucking rate per minute) using lullabies;^(17)^ reduce the frequencies of adverse cardiorespiratory events^(18)^ (defined as the occurrence of apnea &gt; 20 seconds and/or decline in HR to below 100bpm for babies with GA < 34 weeks or below 80bpm for infants &gt; 34 weeks GA), poor sleep-wake cycle,^(19)^ and crying;^(20)^ lower peak HR while feeding, improve sucking, promote faster transition to oral feeding and shorten hospitalization time.^(21)^ One study did not reinforce the beneficial physiological and behavioral effects of lullabies for premature infants. The authors found no significant differences among the intervention (lullaby), placebo and control groups in terms of physiological and behavioral responses.^(22)^ The clinical indicators classified by GRADE are shown in table 1.

#### Olfactory stimulation

**Recommendation:** olfactory stimulation is recommended to prevent apnea using stimulation with vanilla fragrance^(23)^ and to reduce pain using odor stimulation with maternal milk.^(24)^ Olfactory stimulation is not recommended to decrease resting energy using vanilla fragrance,^(25)^ and an unfamiliar odor (vanilla) had no noticeable calming effect on healthy full-term newborns subjected to a painful procedure.^(26)^ The clinical indicators classified by GRADE are shown in table 1.

#### Gustatory stimulation

**Recommendation:** gustatory stimulation using sensorial saturation,^(27)^ maternal milk,^(28)^ assisted suction,^(29,30)^ and sweetened solutions (glucose, sucralose and dextrose) is recommended to reduce pain.^(31-35)^ When sweetened solutions (glucose, sucralose and dextrose) and placebo stimulations^(33-45)^ were compared, the former decreased pain; only one study compared oral sucrose and EMLA® cream,^(37)^ and the combination of sucrose with EMLA® cream had the greatest analgesic effect. The clinical indicators classified by GRADE are shown in table 1.

#### Visual stimulation

**Recommendation:** visual stimulation was included in multimodal SMS rather than unimodal stimulation due to the absence of scientific evidence that met the inclusion criteria of these recommendations.

**Multimodal stimulation**

Multimodal stimulation includes SMS interventions that combine two or more types of sensory stimuli, as follows: tactile-kinesthetic stimulation, therapeutic massage, skin-to-skin control and multisensory stimulation (Figure 1).

#### Kinesthetic tactile stimulation

**Recommendation:** kinesthetic tactile multimodal stimulation is recommended to improve weight gain and reduce hospitalization time,^(46)^ increase parasympathetic activity during sleep,^(47,48)^ improve muscle strength and bone mineralization,^(49)^ improve motor behavior performance,^(50)^ lower bilirubin levels,^(51)^ favor brain electrical activity maturation,^(52)^ favor more mature motor patterns and more regulated and organized behaviors,^(53)^ improve the motor component and shorten hospitalization time,^(54)^ improve fat deposition in preterm newborns;^(55)^ and contribute to strengthening the immunological system and weight gain.^(56,57)^The clinical indicators classified by GRADE are shown in table 1.

#### Massage therapy

**Recommendation:** multimodal SMS using massage therapy is recommended to increase weight gain,^(58-60)^ increase the frequency of defecation episodes,^(61,62)^ decrease transcutaneous bilirubin levels,^(61-63)^ reduce pain scores,^(64)^ and raise the state of alertness after massage.^(65)^ The clinical indicators classified by GRADE are shown in table 1.

#### Skin-to-skin contact

**Recommendation:** SMS using multimodal stimulation with skin-to-skin contact is recommended for newborns on mechanical ventilation,^(66-70)^ reduces pain during painful procedures,^(71-77)^ alleviates stress,^(78,79)^ controls body temperature,^(80)^ is associated with lower newborn salivary cortisol levels,^(79)^ improves the effectiveness of breastfeeding or weight gain,^(81-85)^ and decreases cost of hospitalization.^(86)^

One study^(84)^ did not demonstrate a decline in the salivary cortisol levels of preterm newborns; other investigations^(85,86)^ produced no significant evidence in terms of average daily weight gain. The clinical indicators classified by GRADE are shown in table 1.

#### Multisensory multimodal stimulation

The multisensory stimulation combines different types of stimuli without being necessarily offered simultaneously. Its benefits depend on the maturity of the central nervous system and the sensory subsystems of newborns.^(4,87,88)^

**Recommendation:** multisensory stimulation is recommended to improve the neuromotor score and muscle tone maturation of preterm newborns by applying the “auditory, tactile, visual and vestibular stimulus - ATVV” protocol, improve behavioral organization, raise the frequency of oral behaviors, extend the time spent in the alertness state,^(89,90)^ improve mother-baby interaction with ATVV^(91)^ and increase weight-height growth.^(92)^ The clinical indicators classified by GRADE are shown in table 1.

### Exercises/mobilization

Exercises/mobilization (passive or active-assistive) can be initiated for clinically stable preterm newborns with high risk for bone metabolic disease and GA < 32 weeks and/or birthweight < 1000g.^(93,94)^ The Moyer-Mileur protocol^(93)^ was used in all the studies that met the inclusion criteria of these recommendations.

**Recommendation:** SMS using mobilizations performed by physiotherapists is recommended to increase weight, height and tibial length;^(95)^ raise the speed of tibial ultrasound propagation;^(95,96)^ increase arm circumference,^(97)^ increase bone formation markers and decrease bone resorption markers.^(98,99)^ The clinical indicators classified by GRADE are shown in table 1.

## CONCLUSION

The only sensory motor stimulation modality that has a high degree of scientific certainty was multimodal skin-to-skin stimulation, followed by multisensory stimulation. All modalities have good ratings for pain or stress control. Auditory stimulation stands out for enhancing vital signs, and tactile-kinesthetic massage and multisensory multimodal stimulation stand out for improving weight or sucking. It is recommended that sensory motor stimulation procedures be tailored to the infant’s specific needs and that interventions and be performed by expert professionals.

## APPENDIX 1

**Table 1S:** Studies included for unimodal tactile stimulation recommendations

Author	Type of study	Sample	Interventions	Main outcomes	Main results
Asadollahi et al.^(8)^	Randomized controlled trial	n = 78 NB GA: 30 - 36 weeks Weight: 1,000g - 1,800g Without ventilatory support	Tactile stimulation performed 7 to 10 days after birth; interventions conducted 3 times a day for 5 consecutive days Randomized into 3 groups: Control Group, n = 24: the physiotherapist placed the palm of one hand on the NB's forehead for 15 minutes (fingers on the eyebrows) and the other on the lower abdomen to support the spine and hip Gentle Human Touch Group, n = 27 Massage Group, n = 27	The decrease in stress assessed by urine cortisol level	A decline in cortisol level in the Gentle Human Touch and Massage Group, but significantly higher in the latter
Ramada et al.^(9)^	Prospective controlled trial	n = 40 NB (21 preterm NB and 19 term NB) GA: not informed Weight: not informed	Vital signs and pain level compared before and after therapeutic touch (physiotherapists kept their hands on each region for 3 minutes: head, anterior and posterior chest and regions of the body affected). Total time of 20 - 30 minutes	Assessment of vital sign variability (HR, RR, temperature), Pain assessment according to NIPS	Pain and vital signs declined after intervention (HR, RR)
Bahman Bijari et al.^(10)^	Randomized controlled trial	n = 90 NB GA: 26 - 34 weeks Weight: 1,200g - 2,000g Without ventilatory support	Tactile stimulation performed 7 to 10 days after birth; interventions conducted twice a day for 5 consecutive days Randomized into 3 groups: Control Group, Gentle Human Touch Group and Yakson Group	Assessment of newborns' behavioral status according to the ABSS scale	The newborns improved behaviorally, especially in the sleep phase, which increased in both the Gentle Human Touch and Yakson Groups

NB - newborns; GA - gestational age; HR - heart rate; RR - respiratory rate; NIPS - Neonatal Infant Pain Scale; ABSS - Anderson Behavioral State Scale.

**Table 2S:** Studies included for unimodal auditory stimulation recommendations

Author	Type of study	Sample	Interventions	Main outcomes	Main results
Taheri et al.^(11)^	Double-blind randomized controlled trial	n = 52 NB GA: 33 ± 4 weeks (Intervention Group) GA: 34 ± 4 weeks (Control Group) Weight: not informed	Randomized into 2 groups (n = 26 each): (1) Intervention Group: submitted to auditory stimulation with lullabies, through earphones, at 50 - 60dB, for 20 minutes (2) Control Group: earphones with no music (silence). The two groups were observed for 40 minutes	HR and SpO_2_ assessment	Lullaby (male voice and without music) could significantly reduce heart rate and increase blood oxygen saturation of neonates
Jabraeili et al.^(12)^	Double-blind randomized controlled trial	n = 66 NB GA: 29 - 34 weeks Postnatal age ≥ 3 days, Weight: ≤ 2,800g Without invasive or noninvasive ventilatory support	Randomized into three groups: (1) Lullabies Sung by the Mother Group (n = 21) (2) Brahms Lullaby group (n = 25) (3) Control Group (n = 20): ambient noises. The stimulus was presented at 65 - 70dB, for 15 minutes, between 10am and 7pm, in three consecutive sessions	SpO_2_ assessment	Lullabies sung by the mother and Brahms lullaby music can help to improve SpO_2_ in preterm
Shabani et al.^(13)^	Randomized crossover clinical trial	n = 20 NB, but each infant was studied in two groups of experimental and control. So, totally 40 infants were studied GA: 29 - 36 weeks Birth weight: < 2,500g	Auditory stimulation was performed with Schwartz´ Transitions music (combination of intrauterine sounds of a pregnant woman and a song sung by a female singer), through speakers placed 20cm from the NB´s ear, at 45 - 60dB, for 15 minutes	Physiological responses to pain (HR and SpO_2_), Sleep-wake state, Pain level assessed by the NFCS scale	Playing music is an effective intervention which decreases the heart rate, sleep-wake state scores, and facial expressions of pain
Silva et al.^(14)^	Non-controlled clinical trial	n = 12 NB GA ≤ 36 weeks Weight: not informed Spontaneous breathing	Auditory stimulation performed with classical music twice a day, for 15 minutes, 1 hour after breastfeeding, for 3 consecutive days, totaling 6 interventions The music was played inside the incubator at 45 to 55dB, (total noise = intervention + ambient noises) The speakers were placed outside the incubator, in front of the porthole (which remained open during the intervention), but near the NB's head	Assessment of HR, RR, SpO_2_, BP (systolic and diastolic) and BT	Classical music can change the short-term physiological responses of NBs, HR in one of the sessions, but increased it in the next session; besides, it led to the reduction of HR in two sessions and promoted different variations in SatO2 at when compared to the fifth and sixth session of music therapy
Keidar et al.^(15)^	Randomized crossover prospective clinical trial	n = 12 NB GA: 30 - 37 weeks Tube feeding Weight: not informed	Randomized for auditory stimulation with two types of music (Mozart and Bach) and control (no auditory stimulation), in random sequences, for three consecutive days, The music was always played at midday through speakers placed 30 cm from the NB's ear, at 65 - 70dB, for 30 minutes to one hour after feeding, with the infant in the prone position	Primary outcome was resting energy expenditure, However, vital signs (HR, RR and SpO_2_) were constantly recorded	Auditory stimulation with Mozart music (Mozart Effect) significantly decreased resting energy expenditure
Amini et al.^(16)^	Randomized crossover clinical trial	n = 25 NB GA: 29 - 36 weeks Weight: 1,000g - 2,500g	The NBs were randomly submitted to 2 days of classical music (Mozart), two of lullabies and 2 control days (no music) The stimulus was presented through speakers placed 30cm from the NB's ear, for 20 minutes, with the baby in the supine or prone position	Assessment of vital signs (HR, RR and SpO_2_)	Lullabies lowered HR and RR. These effects continued after exposure, Classical music lowered HR, but the effect disappeared after exposure was discontinued, SpO_2_ did not change with the intervention
Loewy et al.^(17)^	Randomized multicenter controlled trial with blind assessor	n = 272 NB GA ≥ 32 weeks Weight: not informed	All the infants received 3 types of intervention: (1) Lullabies (2) Breathing-like sounds (Ocean disc) (3) Heart beat-like sounds (Gato box) Each child received the three stimulation modalities three times a week (in the morning or afternoon), for two weeks, totaling 6 interventions Auditory stimulation was conducted near the crib or incubator, at 55 - 65dB for 10 minutes The incubator babies received the intervention through an open porthole - Control group: did not receive auditory stimulation	Primary: vital signs (HR, RR and SpO_2_) and activity level, Secondary: feeding behavior (suckings per minute), Sucking behavior (frequency), Sleep pattern, calorie intake	The three interventions positively influenced vital signs, sound and lullaby may improve feeding behaviors and sucking patterns and may increase prolonged periods of quiet-alert states and parent-preferred lullabies, sung live, can enhance bonding, thus decreasing the stress parents associate with premature infant care
Doheny et al.^(18)^	Within-subject experimental study	n = 14 NB GA: 26 to 32 weeks, and at least 27 weeks of age at the time of the study Weight: not informed	Submitted to routine hospital and maternal sounds (voice and heart beats), inside the incubator/crib, at 55 - 60 dBA, 4 times a day, for 30 minutes, Auditory stimulation with maternal sounds within 7 days of birth and continued until NICU discharge	Frequency of adverse cardiorespiratory events (apnea > 20 seconds and/or HR decrease to below 100bpm for babies < 34 gestational weeks or below 80bpm for infants > 34 gestational weeks)	Cardiorespiratory events declined with age., Frequency was lower with exposure to maternal sounds. This effect was significant in infants with GA ≥ 33 weeks, suggesting a therapeutic window
Olischar et al.^(19)^	Randomized controlled trial	n = 20 NB GA: ≥ 32 weeks Weight: not informed Neurologically healthy during the first 6 weeks of life, without ventilatory support	Randomized into 2 groups (n = 10 each): (1) Experimental Group: submitted to Brahms' lullabies, through speakers placed 30cm from the NB's head inside the incubator, at 50 - 55 dBA for 20 minutes (2) Control Group: no auditory stimulation with music	Assessment of aEEG activity:, Background pattern, sleep-wake cycle quality and periods of restful sleep in terms of frequency, duration and minimum and maximum amplitudes	There were no statistically significant intergroup differences; however, the small sample was small
Tramo et al.^(20)^	Randomized prospective controlled trial	n = 13 NB GA: 30 weeks and 6 days to 34 weeks and 3 days Weight: 1,200g - 2,600g at 1 to 35 days of life, without ventilatory support or oxygenation	Randomized into 2 groups: (1) Intervention Group (n = 7): submitted to auditory stimulation with lullabies, through speakers placed 50cm from the infant's head, inside the incubator, at 70 dBA for 10 minutes (2) Control Group (n = 6): no auditory stimulation with music	Assessment of physiological responses (HR, RR and SpO_2_) and behavioral assessment (opening the eyes, head movements and crying) before, during and after heel puncture	In both groups, HR and RR increased during the heel puncture procedure and nearly all the infants cried, During the 10-minute recovery period following heel puncture, HR and crying decreased significantly in the intervention group
Yildiz et al.^(21)^	Quasi-experimental prospective study	n = 90 NB GA: 30 - 34 weeks and exhibiting no sucking reflex Weight: ≥ 1,000g Tolerating enteral diet	Allocated to 3 groups (N = 30/each): (1) Control Group: no intervention (2) Sucking Group: the babies were given pacifiers (3) Lullaby Group: Lullabies were played to the babies through speakers. placed at the infants' feet in the incubator, at 65dB, during force feeding	Assessment of peak HR, RR, and SpO_2_ before, during and after feeding, body weight, sucking success, transition period to oral feeding, and hospitalization time	The pacifier group proceeded to total oral feeding faster, followed by the Lullaby Group, Sucking success was achieved by the pacifier group, followed by the Lullaby Group, The pacifier group had the shortest hospitalization time, followed by the Lullaby Group, There was no difference in RR, SpO_2_ and weight between the 3 groups
Alipour et al.^(22)^	Double-blind randomized placebo-controlled clinical trial	N = 90 NB GA: 28 - 36 weeks and 1 to 25 days of life Weight: not informed	Randomized into 3 groups (n = 30 each): (1) Intervention: auditory stimulation with lullabies, at 50 - 60 dB, through earphones, for 20 minutes, with the NB in the supine position (2) Silence Group (placebo): NB in the supine position with earphones for 20 minutes, without music (3) Control Group: no intervention; routine care	Physiological responses (HR, RR and SpO_2_), Behavioral responses assessed by the BSI scale	No significant intergroup differences in terms of physiological and behavioral responses,

NB - newborns; GA- gestational age; HR - heart rate; SpO_2_ - blood oxygen saturation; NFCS - Neonatal Facial Coding System; RR - respiratory rate; BP - blood pressure; BT - body temperature; NICU - neonatal intensive care unit; aEEG - amplitude-integrated electroencephalogram; BSI - Behavioral State Instrument.

**Table 3S:** Studies included for unimodal olfactory stimulation recommendations

Author	Type of study	Sample	Interventions	Main outcomes	Main results
Edraki et al.^(23)^	Randomized prospective controlled clinical trial	n = 36 premature NB GA: 33 - 35 weeks Weight: < 2,500g	Randomized into two groups (18 NB in each group): Control Group: submitted to routine care Intervention Group: olfactory stimulation with vanillin solution	Frequency of apnea episodes, arterial blood oxygen saturation and HR	There was a reduction in apnea episodes in the group submitted to vanillin solution, There was no change in vital signs between groups
Baudesson de Chanville et al.^(24)^	Double-blind randomized placebo-controlled clinical trial	n = 33 NB GA: 30 - 36 weeks Weight: > 1,500g	Randomized into two groups: Control Group: 17 NB submitted to routine care Intervention Group: 16 NB Olfactory stimulation with maternal milk 10 days after birth	Pain assessment using PIPP and changes in vital signs (HR & SPO2) of NBs submitted to venipuncture	Significant pain reduction without changes in vital signs
Marom et al.^(25)^	Randomized prospective controlled clinical trial	n = 20 NB GA: 32 - 35 weeks Weight: not informed Without ventilatory support	Olfactory stimulation with vanillin odour exposureversus no vanillin odour exposure for 2 consecutive days	Metabolic rate evaluated through indirect calorimetry; oxygen (VO_2_) and carbon dioxide (VCO_2_) consumption	There was no difference between the two groups in terms of resting energy expenditure,
Romantsik et al.^(26)^	Randomized prospective controlled clinical trial	n = 69 NB GA: ≤ 37 weeks Weight: 3,000g - 4,000g	Randomized into two groups: Control Group: 30 NB submitted to water scent (7cm from the nose) for 3 minutes Intervention Group: 39 NB olfactory stimulation with vanilla scent (7cm from the nose) for 3 minutes	Responses to painful procedures with vanilla and water scents were measured using two scales, the NFCS and BIIP, along with crying duration and hand movements	There were no statistically significant differences between stimulation with vanilla scent compared to water use for the reduction of pain after painful procedures

NB - newborn; GA - gestational age; HR- heart rate; PIPP - premature infant pain profile; SpO_2_ - blood oxygen saturation; VO_2_ - maximum oxygen volume; VCO_2_ - maximum carbon dioxide volume; NFCS - Neonatal Facial Coding System; BIIP - Behavioural Indicators of Infant Pain.

**Table 4S:** Studies included for unimodal gustatory stimulation recommendations

Author	Type of study	Sample	Interventions	Main outcomes	Main results
Bernardini et al.^(27)^	Randomized prospective controlled clinical trial	n = 28 NB GA: 30 - 35 weeks Weight: not disclosed	Glucose solution associated to no nutritive suction versus sensorial saturation (14 each) comprising a 6-step protocol: Keep the NB in lateral position Maintain eye contact Massage face and back Talk to the baby gently but firmly Allow child to smell the fragrance of a baby oil (Babygella, Guieu Labs) on the therapist's hands place 10% glucose on the baby's tongue	Assess pain level during venipuncture using the PIPP scale	Sensorial saturation ameliorates the quality of life in NICU and reduces the pain threshold perceived by newborn
Bueno et al.^(28)^	Randomized prospective controlled clinical trial	n = 113 NB GA: 34 - 36 weeks Weight: ≥ 2,000g	Stimulation with maternal milk versus glucose	Evaluate pain level by comparing stimulation with maternal milk versus glucose, before NB heel puncture, using the PIPP	No statistically significant differences were found between the interventions
Cignacco et al.^(29)^	Randomized prospective multicenter controlled clinical trial	n = 71 NB GA: 24 - 32 weeks Weight: not disclosed	Stimulation with sucrose, facilitated tucking, and a combination of both interventions during heel puncture and collected during the first 14 days of their NICU stay	Assess pain level by comparing sucrose versus assisted suction, during heel puncture of NBs, using the BPNS score (combination of items that evaluate physiological and behavioral status)	Sucrose, regardless of association, showed better efficacy in reducing pain
Mekkaoui et al.^(30)^	Randomized prospective controlled clinical trial	n = 125 NB GA: 28 - 37 weeks Weight: not disclosed	Randomized into five non-pharmacological interventions: Oral glucose stimulation Non-nutritive sucking Oral administration of glucose 30%; associated with pacifier suction Oral administration of glucose 30%; associated with formula milk Oral administration of age-appropriate formula milk	Assess pain level during heel puncture using the DAN scale	Among the five groups, NB who received 30% glucose, artificial milk, or sucking a pacifier showed better results
Ou-Yang et al.^(31)^	Randomized prospective controlled clinical trial	n = 123 NB IG: < 37 weeks Weight: not disclosed	Stimulation with distilled water (n = 48) versus 25% glucose water (n = 50) versus maternal milk (n = 62)	Assess pain level during heel puncture using the PIPP scale	Stimulation with maternal milk decrease pain level
Costa et al.^(32)^	Randomized prospective controlled clinical trial	n = 124 NB GA: ≤ 32 weeks Weight: ≤ 1,500g	Administration of a 1mLdose of 25% glucose solution 2 minutes before and immediately after the first retinopathy of prematurity (ROP) eye examination	Evaluate pain intensity during the first eye examination for retinopathy of prematurity in NB using the NPAS scale	Glucose decreases the pain level of the first eye examination for retinopathy of prematurity
Nimbalkar et al.^(33)^	Randomized prospective controlled clinical trial	n = 104 NB GA: > 28 weeks Weight: not disclosed	One group received lingual dextrose before orogastric tube placement and the other received a placebo	Evaluate pain reduction after orogastric tube placement, with lingual administration of 25% dextrose, using the PIPP scale	Lingual dextrose administration decreases pain level compared to the placebo group
Pandey et al.^(34)^	Randomized prospective controlled clinical trial	n = 105 NB IG: < 37 weeks (< 168 hours of life) Weight: not disclosed	Stimulation with 24% sterile sucrose solution (n = 53) versus distilled water (n = 52), both administered orally 2 minutes before orogastric tube placement	Evaluate pain level following orogastric tube placement using the PIPP scale	Sucrose reduced pain level compared to the placebo group
Sahoo et al.^(35)^	Randomized prospective controlled clinical trial	n = 160 NB GA: > 34 weeks Weight not disclosed	Three groups randomized into: Stimulation with maternal milk (n = 50) 25% dextrose (n = 62) Sterile water (n = 48)	Assess pain level after venipuncture using the PIPP scale and changes in HR, SpO_2_ and duration of crying	Dextrose and maternal milk reduced pain level compared to the placebo group
Scaramuzzo et al.^(36)^	Randomized prospective controlled clinical trial	n = 158 NB GA: > 37 weeks Weight: not disclosed	Randomized into two groups: stimulation with oral sucrose (n = 82) versus wrapping (n = 76)	Evaluate spontaneous fluctuations in skin conductance to measure pain and to compare oral sucrose with non-apharmacological analgesic packaging	Oral sucrose is more effective in reducing pain level
Al Qahtani et al.^(37)^	Randomized prospective controlled clinical trial	n = 90 NB GA: > 38 weeks Weight: not disclosed	Stimulation with EMLA cream (n = 30) versus oral sucrose (n = 30) versus combination of EMLA cream and oral sucrose (n = 30)	Assess pain level comparing EMLA use and oral sucrose in NBs during circumcision	The combination of sucrose with EMLA cream has the greatest analgesic effect
Dilli et al.^(38)^	Randomized prospective controlled clinical trial	n = 64 NB GA: 28 - 37 weeks Weight: not disclosed	Stimulation with sucrose on pacifier (n = 32) versus sucrose-free pacifier versus sterile water (n = 32) 30 seconds before eye examination	Evaluate effectiveness of oral sucrose combined with non-nutritive sucking to reduce pain level	Sucrose combined with non-nutritive sucking reduces pain level during eye examinations
Ravishankar et al^(^39^)^	Randomized prospective controlled clinical trial	n = 150 NB GA: > 34 weeks Weight: not disclosed	Randomization into 3 groups: stimulation with 25% dextrose (n = 50) versus 10% dextrose (n = 50) versus distilled water (n = 50), 2 minutes before nasogastric tube placement	Assess pain level using the PIPP scale, crying duration, and changes in heart rate	25% oral dextrose reduces pain level before nasogastric tube placement
Suhrabi et al.^(40)^	Randomized prospective controlled clinical trial,	n = 90 NB GA: > 37 weeks Weight: not disclosed	Randomization into three groups: stimulation with glucose (n = 30) versus oral sucrose (n = 30) versus placebo (n = 30), 2 minutes before vaccination	Assess pain intensity before the Hepatitis B vaccination using the NIPS scale	Both glucose and sucrose are equally effective in decreasing pain when administered before the Hepatitis B vaccination
Uzelli et al.^(41)^	Randomized prospective controlled clinical trial	n = 80 NB GA: > 33 weeks Weight: not disclosed	Oral glucose administration (n = 40) versus control group (n = 40) 2 minutes before intramuscular injection	Assess pain intensity using the NIPS scale	Oral glucose, even when used in low amounts, is effective in reducing pain before intramuscular injection
Kataria et al.^(42)^	Randomized prospective controlled clinical trial	n = 24 NB GA: > 31 weeks Weight not disclosed	Administration of 2mL dextrose (n = 12) versus control group (n = 12 PTNBs) 2 minutes before retinopathy of prematurity corrective laser surgery	Assess pain intensity using the PIPP scale before and 30 seconds after starting the laser treatment	A single dose of oral dextrose did not significantly reduce pain during laser treatment in premature neonates
Tutag Lehr et al.^(43)^	Randomized prospective controlled clinical trial	n = 56 NB IG: > 37 weeks (≤ 7 days of life) Weight: not disclosed	Randomized into 2 groups: stimulation with 24% sucrose (n = 29) versus sterile water (n = 27) 10 minutes before heel puncture	Assess pain and blood flow, in NB under the effect of oral sucrose, using laser doppler and NIPS scale	Blood flow and pain after heel puncture were less in NB who received 24% sucrose
Vezyroglou et al.^(44)^	Randomized prospective controlled clinical trial	n = 32 NB GA: < 37 weeks Weight: > 1,500g	Randomized into 2 groups: stimulation with glucose (n = 16) versus placebo group (n = 16), 3 minutes before oropharyngeal aspiration	Evaluate pain level using the PIPP scale	Pain level did not differ between the two groups
Medeiros et al.^(45)^	Randomized prospective controlled clinical trial	n = 90 NB GA: 28 - 36 weeks Weight: not disclosed	Randomization into 2 groups: stimulation with water (n = 46) versus sucrose (n = 44) to analyze specific hand-to-mouth and hand sucking behaviors	Assess motor behavior and behavioral status of NB by observing hand-to-mouth and hand sucking behavior	Oral stimulation had a positive influence on hand-mouth coordination, regardless of the stimulus (water or sucrose)

NB - newborn; GA - gestational age; PIPP - Premature Infant Pain Profile; NICU - neonatal intensive care unit; BPNS - Bernese Pain Scale Neonatal Scale; DAN - Douleur Aigue Nouveau ne scale; ROP - retinopathy of prematurity; NPAS -Neonatal Pain, Agitation and Sedation; HR - heart rate; SpO_2_- blood oxygen saturation; NIPS - Neonatal Infant Pain Score; PTNB - preterm newborn.

**Table 5S:** Studies included for multimodal tactile-kinesthetic stimulation recommendations

Author	Type of study	Sample	Interventions	Main outcomes	Main results
Ahmed et al.^(46)^	Prospective quasi-experimental clinical trial	n = 151 NB GA: 30 - 37 weeks Weight: 1,000g - 1,200g Without ventilatory support	Randomized into two groups: - Control Group: 76 NB submitted to routine care - Intervention Group: 75 NB submitted to tactile-kinesthetic stimulation for 15 minutes (3 times a day for 7 consecutive days)	Weight gain and length of hospital stay	Tactile kinesthetic group had greater weight gain and shorter length of hospital stay compared to the control group
Smith et al.^(47)^	Randomized prospective double-blind placebo-controlled clinical trial	n = 21 NB GA: 30 - 31 weeks Weight: 1,500g	Randomized into two groups: Control Group: 11 NB, therapists stood beside the incubator for 20 minutes twice a day Intervention Group : 10 NBs submitted to 20 minutes of massage + kinesthetic movement (Moyer-Mileur) twice a day	Autonomic nervous system function during sleep stages and assistive care, measured by HRV after 2 weeks of massage therapy performed twice a day	There was no difference in HRV between intervention group and control group
Smith et al.^(48)^	Randomized prospective single-blind controlled clinical trial	n = 21 NB GA: 29 - 32 weeks Weight: 1,500g	Randomized into two groups: Control Group: 20 NB, therapists stood beside the incubator for 20 minutes twice a day Intervention Group: 17 NB, 20 minutes of massage + kinesthetic movement (Moyer-Mileur) twice a day for 4 weeks	Heart rate variability through ECG	Massage improved the sample's autonomic nervous system function
Haley et al.^(49)^	Randomized prospective single-blind controlled clinical trial	n = 40 NB GA: < 37 weeks Weight: 1,500g - 1,600g	Randomized into two groups: Control Group: 20 NB are placed in a supine position without tactile stimulation and without kinesthetic TKS Group: movement tactile/kinesthetic stimulation: 20 NB, 20 minutes of massage twice daily, 6 days per week for 2 weeks TKS = (1) legs from top of thighs to ankles and feet, (2) chest over ribcage, (3) shoulders to hands, (4) head from crown to neck and including face, (5) back from neck to waist (performed with infant remaining in supine position)	Increase in anthropometry; in the quantitative measurement of the ultrasound-assessed tibial speed of sound; urine and blood markers	TKS improved bone strength; bone metabolism biomarkers suggest improved bone mineralization in the massage group.
Aliabadi et al.^(50)^	Randomized prospective controlled clinical trial	n = 40 NB GA: 29 - 32 weeks Weight: > 1,500g and < 2,499g	Randomized into two groups: Control Group: 20 NB; Intervention Group: 20 NB submitted to 15-minutes interventions divided into three phases: 5 minutes massage + 5 minutes. TKS + 5 minutes massage (field protocol), 3 times daily for 10 consecutive days	Behavioral state assessment	The group that received TKS showed better motor and regulation state
Chen et al.^(51)^	Randomized prospective controlled clinical trial	n = 42 NB GA: 37 - 41 weeks Weight: 2,800g to 3,600g	Randomized into two groups: Control Group: 22 NB Intervention Group: 20 NB submitted to 15 to 20-minute interventions (touch therapy by field protocol), performed twice daily for 5 consecutive days	Assessment of bowel movement frequency, measurement of transcutaneous jaundice and serum bilirubin levels	The group that received massage (touch therapy by field protocol), showed lower bilirubin levels
Guzzetta et al.^(52)^	Randomized prospective controlled clinical trial , ,	n = 20 NB GA: 30 - 33 weeks Stable hospitalized NB Weight: between the 25th and 75th percentile	Randomized into two groups: Control Group: 10 NB Intervention Group: 10 NB submitted to 15-minutes interventions divided into two phases: 10 minutes massage + 5 minutes. Kinesthetic movement, performed 3 times daily for 5 consecutive days over a period of 2 weeks (twoday interval between weeks)	ECG assessment of brain electrical activity	Massage favored a maturation process of brain electrical activity similar to that observed in utero
Ferreira et al.^(53)^	Randomized prospective controlled clinical trial	n = 32 NB GA: 31 - 33 weeks Weight: < 2,500g	Randomized into two groups: Control Group: 16 NB Intervention Group 16 NBs submitted to TKS	Assessment of behavioral state	TKS contribute towards adjustment and self-regulation of behaviour
Ho et al.^(54)^	Randomized prospective controlled clinical trial	n = 20 NB GA: < 34 weeks Weight: < 1,500g	Randomized into two groups: Control Group: 12 NB received similar duration of light still touch Intervention Group: 12 NB received massage therapy starting at 34 weeks post-conceptional age (15 minutes daily, 5 days/week for 4 weeks)	Assessment of motor performance using the TIMP scale	Improvement in motor performance (NB with low TIMP score before intervention); shorter length of hospital stay for intervention group
Moyer-Mileur et al.^(55)^	Randomized prospective single-blind controlled clinical trial	n = 44 NB GA: 29 - 32 weeks Weight: 1,300g - 1,800g	Randomized into two groups: Control Group: 22 NB, therapists stood beside the incubator twice a day Intervention Group: 22 NBs submitted to interventions of 20 minutes, divided into massage + TKS, twice daily for 6 consecutive days over a period of 4 weeks (Moyer-Mileur Protocol)	Evaluate weight gain and fat deposition	massage may improve body fat deposition, and in turn growth quality, of preterm infants in a sex-specific manner
Ang et al.^(56)^	Randomized prospective controlled clinical trial	n = 120 NB GA: 30 weeks Weight: 600g - 1,800g	Randomized into two groups: Control Group: 62 NB Intervention Group: 58 NB submitted to interventions of 5 to 15 minutes, divided into three phases: 5 minutes. massage + 5 minutes. TKS + 5 minutes. massage (field protocol) 3 times daily for a minimum of 5 days (maximum of 4 weeks or until hospital discharge)	Assessment of massage effects on the immune system	Increase in white blood cells and greater weight gain in the intervention group
Diego et al.^(57)^	Randomized prospective controlled clinical trial	n = 30 NB GA: 28 - 32 weeks stable hospitalized NB Weight: 1,000 - 1,500g	Randomized into two groups: Massage Group: 15 NB submitted to massage movements three times a day for 10 minutes over 5 days TKS Group: 15 NB submitted to limb flexion /extension 3 times a day for 10 minutes over 5 consecutive days (field and Moyer-Mileur adapted protocol)	Assessment of vagal activity (ECG in the first week of intervention) and calorie consumption	Increase in weight gain in the intervention group

NB - newborn; GA - gestational age; HRV - heart rate variation; ECG - electrocardiogram; TKS - tactile/kinesthetic stimulation group; TIMP - Test of Infant Motor Performance.

**Table 6S:** Studies included for multimodal massage stimulation recommendations

Author	Type of study	Sample	Interventions	Main outcomes	Main results
Kumar et al.^(58)^	Randomized controlled clinical trial	n = 44 GA: < 35 weeks Weight: < 1,800g	Two groups: 23 in the control group 25 submitted to massage therapy using oil on both shoulders starting from the neck with baby in prone position, then the upper back to hip area followed by the upper and lower limbs, one at a time in supine position for 10 minutes, 4 times daily over a period of 28 consecutive days	Weight gain; serum triglyceride level; length and head circumference	The massage group presented weight gain after 28 days and decreased weight loss within the first 7 days. There was no statistical difference regarding the other outcomes
Seaedi et al.^(59)^	Randomized controlled clinical trial	n = 121 GA: < 37 weeks Weight: not disclosed	Three groups: Massage therapy group with oil (n = 40) Massage therapy (n = 40) Control group (n = 40) 5 minutes of massage 4 times a day for 7 days	Weight gain	There was greater weight gain in the oil massage therapy group compared to the other 2 groups
Fallah et al.^(60)^	Randomized controlled clinical trial	n = 57 GA: 33 - 37 weeks	Two groups: Group 1: only moderate pressure massage therapy (n = 17) Group 2: massage therapy with moderate pressure and oil (n = 17)	Weight gain on day 14, 1 and 2 months after the interventions	The group submitted to massage oil therapy exhibited greater weight gain in all assessments compared to the other group
Basiri Moghadam et al.^(61)^	Randomized controlled clinical trial,	n = 40 GA: 34 - 36 weeks Weight: not disclosed	Two groups: 20 controls 20 underwent massage therapy. Type of massage therapy not disclosed. Intervention time: 20 minutes twice a day for 4 consecutive days	Number of bowel movements; transcutaneous bilirubin level	The massage therapy group had a higher number of bowel movements and lower transcutaneous bilirubin levels
Lin et al.^(62)^	Randomized controlled clinical trial	n = 56 GA: 30 - 40 weeks Weight: not disclosed	Two groups: Control Group (n = 29) Massage Therapy group (n = 27)	Length of hospital stay; weight gain; increased bowel movement frequency; microbilirubin level	There was an increase in bowel movement frequency and a decrease in bilirubin levels in the massage group; there was no difference between the groups for the other outcomes
Dalili et al.^(63)^	Randomized controlled	n = 50 GA: 36 - 40 weeks Weight: not disclosed	Two groups: Control Group (n = 25) Massage Group (n = 25)	Increased bowel movement frequency; transcutaneous bilirubin level	There was a decrease in bilirubin levels in the massage group. There was an increase in bowel movement frequency in the control group on day 1. On the subsequent days, there was no difference between the two groups
Chik et al.^(64)^	Randomized controlled clinical trial	n = 80 GA: 30 to 40 weeks	Two groups: Control Group (n = 40) Massage Group (n = 40): upper limb massage before and after venipuncture	Pain level reduction assessed by PIPP	There was a reduction in pain score in the group submitted to massage therapy
Yates et al.^(65)^	randomized cross-over study	n = 23 GA: 32 - 48 weeks Weight: not disclosed	Two groups: Massage therapy the first day (n = 13) Massage therapy the second day (n = 10)	Massage therapy can be used as an adjunct intervention to induce sleep	Massage therapy did not induce sleep immediately after massage and infants are more wakeful following massage therapy

GA - gestational age; PIPP - Premature Infant Pain Profile.

**Table 7S:** Studies included for multisensory skin-to-skin stimulation recommendations

Author	Type of study	Sample	Interventions	Main outcomes	Main results
Azevedo et al.^(66)^	Quasi-experimental study design	n = 43 GA: > 29 weeks Weight: not disclosed Hemodynamically stable, intubated receiving MV	A group evaluated longitudinally on 3 occasions: before, during and after the procedure for a duration of 90 minutes	Procedure safety, Variables measured: HR, SpO_2_, FiO2, mean arterial blood pressure, and temperature	Changes in the variables studied were not clinically significant (< 5% from baseline) although statistically significant. Skin-to-skin contact is a safe procedure for NBs receiving MV
Carbasse et al.^(67)^	Quasi-experimental study design study	n = 96 GA: 24 - 33 weeks Weight: > 500g Vulnerable preterm infants	Vital signs, body temperature, and oxygen requirement data were prospectively recorded by each infant's nurse before (baseline), during (3 time points), and after their first skin-to-skin contact. 17 clinically stable NBs receiving MV, 49 with nasal CPAP and 30 spontaneously breathing room air	Safety and physiological effectiveness of the procedure, Evaluate the impact of the respiratory support. Variables measured: HR, SpO_2_, FiO2, transcutaneous partial pressure of carbon dioxide (TcPCO_2_), and temperature	Changes in the variables studied were statistically significant , Skin-to-skin contact is an effective and safe procedure for vulnerable preterm infants
Karlsson et al.^(68)^	Quasi-experimental study design	n = 27 GA = 22 to 26 weeks Weight: not disclosed	Infants' skin temperature and body temperature, ambient temperature, and relative humidity were measured during pretest (in incubator), test (during SSC), and posttest (in incubator) periods	Assessment of the NICU's thermal balance and physical care environment during skin-to-skin, Variables studied: Relative humidity and air temperature in the incubator and skin-to-skin environment; corporal temperature; skin temperature; evaporimetry (transepidermal water loss and insensible water loss)	Early skin-to-skin initiation allows thermoregulation to occur in even the smallest NBs receiving intensive care, including mechanical ventilation , Skin-to-skin is an important and safe care mode for extreme PTNBs, even with mechanical ventilation
Lorenz et al.^(69)^	Prospective observational non-inferiority study	n = 40 GA < 33 weeks Weight: > 800g Receiving ventilatory support (ETT, CPAP or HFNC)	rcO2 was measured using near-infrared spectroscopy. Ninety minutes of skin-to-skin contact, with infants in incubators acting as their own control	Evaluate rcO2 on two occasions (skin-to-skin contact, and incubator care). Secondary outcomes included physiological parameters (SpO_2_, HR, FiO2, cFTOE and AT) evaluated on both occasions and divided by ventilatory support modality.	Cerebral oxygenation and other physiological measurements in ventilated preterm infants did not differ between skin-to-skin contact, and incubator care
Park et al.^(70)^	Prospective clinical trial,	n = 31 GA: 25 - 32 weeks Weight: 760g - 1,740g	Two groups submitted to skin-to-skin contact: 25 - 28 weeks (n = 11) and 29 - 32 weeks (n = 20)	Determine the clinical characteristics and safety of skin-to-skin contact according to GA, Physiological parameters were evaluated longitudinally for 60 minutes (15 minutes before, 30 minutes during and 15 minutes after the procedure). , Variables studied: HR, RR, SpO_2_, blood pressure, temperature	Changes in the variables studied were not clinically significant (< 5% from baseline) although some were statistically significant. , At the same post-menstrual age, the lower GA group showed greater thermoregulation maturation compared to the higher GA group, Skin-to-skin contact is a safe procedure for PTNBs even while receiving ventilatory support
Okan et al.^(71)^	Randomized controlled clinical trial, ,	n = 107 GA: > 37 weeks Age between 24 and 48 hours of life Weight: not disclosed Healthy NB	Three groups: Skin-to-skin contact group (N = 35) with maternal breast feeding; n = 36 skin-to-skin contact, n = 36 placed in the crib, evaluated before, during and after the painful stimulus	Evaluate the effectiveness of skin-to-skin contact for pain reduction during heel puncture in FTNBs, Assess whether the combination of skin-to-skin contact with breastfeeding provides greater analgesia than skin-to-skin contact alone, Variables studied: Crying time after painful stimulus. Secondary outcomes: HR, SpO_2_	HR, SpO_2_, and crying duration was significantly lower in the groups skin-to-skin and skin-to-skin contact with breastfeeding, compared to NBs in the crib who underwent the painful procedure, Skin-to-skin contact before, during, and after a painful stimulus promotes a reduction in physiological and behavioral responses to pain in healthy FTNBs, The combination of skin-to-skin contact with breastfeeding promotes an analgesic effect similar to skin-to-skin contact only
Saeidi et al.^(72)^	Randomized controlled clinical trial	n = 60 GA: healthy fullterm NBs Weight: not disclosed	Two groups: skin-to-skin contact for 30 minutes (n = 30) and control (wrapped in blanket and placed next to mother, n = 30)	Evaluate the effect of skin-to-skin contact on pain intensity using the NIPS scale in healthy NBs undergoing a painful procedure (vaccination), Secondary outcomes: HR, SpO_2_ and crying duration	Mean pain intensity during the procedure, and 3 minutes after, was significantly lower in the skin-to-skin contact group, Kangaroo care may be used to decrease pain intensity in newborns undergoing painful procedures
Cong et al.^(73)^	Randomized crossover clinical trial	n = 28 GA: 28 - 32 weeks Weight: not disclosed < 14 days of life in heated incubator	Three groups randomized into different procedure sequences, evaluated on 6 occasions: skin-to-skin for 30 minutes, skin-to-skin for 15 minutes, and standard care	Skin-to-skin effect (duration of 30 and 15 minutes) on the autonomic pain response of PTNBs subjected to heel puncture compared to standard care. , Variables studied: HR variability, behavioral state	Both skin-to-skin contact durations, before and during heel puncture, promote prolonged restful sleep after puncture. , Kangaroo care has a significant effect on reducing autonomic pain responses in preterm infants. The findings support that KC is a safe and effective pain intervention, in the neonatal intensive care unit
Nimbalkar et al.^(74)^	Randomized controlled clinical trial	n = 100 term and late preterm Weight: > 1,800g	Two groups: Intervention Group: SSC at 30 minutes to 1 hour after delivery and continue for as long as possible in the first 24 hours with each session lasting for minimum 60 minutes, Control Group, after providing routine care under radiant warmer, NBs were kept clothed (including head cap) and covered with blanket with their mother (bedding in) for first 48 hours	Temperature and heart rate	The incidence of hypothermia in conventional care was significantly higher as compared with the SSC
Chidambaram et al.^(75)^	Randomized crossover clinical trial	n = 100 GA: 32 - 36 weeks Weight: < 2,500g Hemodynamically stable without dependence on oxygen.	Two groups: Control Group (n = 50) Skin-to-Skin Contact Group (n = 50)	PIPP pain scale assessment 15 minutes before, 15 and 30 minutes after heel puncture	PIPP scores at 15 and 30 minutes after puncture were significantly lower in the skin-to-skin contact group compared to the control group, Skin-to-skin contact is effective in reducing pain in PTNBs subjected to heel puncture
Gao et al.^(76)^	Randomized controlled clinical trial	n = 75 GA: < 37 weeks Weight: 2,030g	Two groups: Control group (n = 37) Skin-to-Skin Group (n = 38)	Evaluate the effectiveness of 30 minutes of skin-to-skin contact on behavioral and physiological responses of PTNBs undergoing heel punctures. During the first puncture procedure, all NBs were kept in the incubator. In the other three procedures, the NBs were randomized into skin-to-skin contact or standard incubator care; evaluators were blinded to the purpose of the study. , Variables studied: facial expression, crying, and HR in four heel puncture procedures	HR was significantly lower, crying and grimacing duration was significantly shorter (from time of heel puncture to recovery) across repeated heel puncture procedures in the skin-to-skin group compared to the control group, The effect of repeated Kangaroo Mother Care analgesia remains stable in preterm infants over repeated painful procedures
Choudhary et al.^(77)^	Quasi-experimental crossover single-blind clinical trial	n = 140 Weight: > 1,000g	One group (n = 140): each NB was its own control GA < 37 weeks All NBs were divided into gestational age: 28 - 30 weeks (n = 80); 30 - 34 weeks (n = 60) and birth weight: 1,000g - 1,500 g (n = 88); 1,500 - 2,500g (n = 52)	Assessment of PIPP during heel puncture, HR, SpO_2_, crying duration and recovery time,	The effect of skin-to-skin contact was statistically significant in the PTNBs (30 - 34 weeks) and very low weight (1,000 - 1,500g) groups, SpO_2_ drop was lower (36% reduction) in the skin-to-skin contact group than in conventional care, Crying duration was shorter in the skin-to-skin contact group than in conventional care, with a statistically significant difference, PIPP scores were significantly lower with skin-to-skin contact, Implementing skin-to-skin contact is a safe method of helping physiological and behavioral stability in PTNBs
Kaffashi et al.^(78)^	Randomized quasi-experimental crossover clinical trial	n = 134 NBs (8 PTNBs) Received 8 weeks of skin-to-skin contact.	Three groups: PTNBs received skin-to-skin contact for 8 weeks (16 EEG recordings during sleep, n = 8) compared to two groups (nN = 126): one group of PTNBs with corrected gestational age and another of FTNBs	Neurophysiology maturation of the neonatal brain by quantifying temporal characteristics (regularity and predictability) of sleep EEG signals, , ,	The group of PTNBs that received skin-to-skin contact exhibited more complex EEG signals compared to PTNBs of the same gestational age, Discriminatory analyses show that PTNBs who received skin-to-skin contact (at 40 weeks corrected age) exhibit patterns closer to FTNBs than PTNBs not submitted to this intervention, at the same gestational age.
Neu et al.^(79)^	Randomized controlled clinical trial	n = 79 GA: 32 - 35 weeks Weight: not disclosed	Three groups: Control Group (n = 24) Skin-to-Skin Contact Group (n = 29) Group Wrapped in Blanket (n = 26	Evaluate coregulation in salivary cortisol between mother and NB; coregulation defined as progressive reduction in the absolute difference between mother and NB cortisol levels during each 60-minute session of skin-to-skin contact, Variable studied: coregulation of salivary cortisol between mother and NB	Decreased cortisol levels in mothers and NBs suggesting that skin-to-skin contact caused a decline in stress hormone levels, There was no significant difference in coregulation between the groups, nonstressful situations, co-regulation in salivary cortisol may not differ based on holding method
Srivastava et al.^(80)^	Randomized controlled clinical trial	n = 240 GA: any age Weight > 2,500g Skin-to-skin in the first 30 minutes of life	Two groups: Control Group (n = 118) Skin-to-Skin Contact Group (n = 122) Variables studied: breastfeeding effectiveness, 6-week breastfeeding status, maternal satisfaction, thermoregulation, weight loss at hospital discharge and at first follow-up, and morbidity	Evaluate the impact of early skin-to-skin initiation on breastfeeding effectiveness and maternal satisfaction in relation to perceived NB breastfeeding status at hospital discharge, Secondary outcomes: related to neonatal well-being (thermoregulation in the immediate postpartum period, NB weight parameters and morbidities during the first six weeks of life)	Skin-to-skin contact contributed to greater breastfeeding effectiveness, more infants being exclusively breastfed at the first follow-up and at 6 weeks, greater maternal satisfaction, better immediate postpartum temperature gain, lower weight loss at hospital discharge and first follow-up, and lower morbidity when compared to the control group
Jayaraman et al.^(81)^	Randomized controlled clinical trial, ,	n = 160 Weight: 1,000g - 1,800g	Two groups: early skin-to-skin contact initiated within the first 4 days of life (n = 80); late skinto- skin contact, initiated after complete stabilization, defined as absence of respiratory support and intravenous fluids (n = 80)	Evaluate the effects of early skin-to-skin contact initiation on exclusive breastfeeding, growth, mortality and morbidity compared to late initiation (during hospitalization and after hospital discharge) in low-weight NBs	The early skin-to-skin contact group had higher proportion of exclusive breastfeeding, higher breastfeeding rate during hospitalization, and a higher proportion of exclusive breastfeeding up to one month after hospital discharge, The incidence of apnea and recurrent apnea requiring ventilation was significantly reduced in the early skin-to-skin contact group, There was no significant difference in mortality, morbidity, and growth during hospitalization and after hospital discharge
Nagai et al.^(82)^	Randomized controlled clinical trial,	n = 73 Weight: < 2,500g Age: < 24 hours Relatively stable clinical conditions	Two groups: early skin-to-skin contact within the first 24 hours of life (n = 37). Control: conventional care performed initially with skin contact after 48 - 72 hours of life (n = 36).	Evaluate the effectiveness of early skin contact initiation for relatively stable low-weight NBs in a resource-constrained country	There were no differences in the incidence of morbidity, Weight loss from birth up to 24 hours (and up to 48 hours) of life was significantly lower in the early skin-to-skin contact group compared with the control group, The occurrence of adverse effects and length of hospitalization did not differ between the groups
Sharma et al.^(83)^	Randomized controlled clinical trial	n = 141 Weight: < 1,100g Clinically stable	Two groups: skin-to-skin contact (n = 71) and conventional care (n = 70) when NBs reached 1,150g.	Assess weight gain (g/day) from start of randomization to full-term (40 weeks), Variables studied: weight gain (g/day), Secondary outcomes: weight, length and head circumference at 40 weeks; intra-hospital weight gain (g/day), length (cm/week) and head circumference (cm/week) following randomization; breastfeeding rates at hospital discharge and at full-term age; neonatal ICU readmissions (level III or intermediate care unit)	Average weight gain, as well as weight, length and head circumference at term corrected age were comparable in both groups, There was a significant reduction in hospitalization time in the conventional care group and a significant increase in weight gain before discharge in the skin-to-skin contact group
Mitchell et al.^(84)^	Randomized controlled clinical trial, ,	n = 38 GA: 27 - 30 weeks Weight: < 1,311g Receiving ventilatory support (MV, CPAP or nasal cannula)	Two groups: Control Group (n = 19) Skin-to-Skin Contact Group (n = 19)	Determine if stress on PTNBs (measured by salivary cortisol levels) decreases after 5 days of skin-to-skin contact compared to 5 days in standard care, Determine if skin-to-skin provides sustained pain relief following the period of contact, Salivary cortisol was collected and evaluated on the 5th and 10th day of life, PIPP pain score was measured during routine tracheal and nasal aspiration because such procedures are considered painful,	Skin-to-skin contact did not affect basal salivary cortisol levels in PTNBs compared to standard care , Salivary cortisol levels decreased in both groups between the 5th and 10th day of life, demonstrating that the day of life variable should be considered when salivary cortisol is evaluated in PTNBs, Skin-to-skin contact did not affect PIPP scores after aspiration, as NBs were not in skin-to-skin contact during the procedure
Ghavane et al.^(85)^	Randomized controlled clinical trial	n = 140 Weight: < 1,500g Hemodynamically stable; tolerating spoon feeding of 150mL/kg /day	Two groups: skin-to-skin contact (n = 71) and conventional care (n = 69)	Evaluate the effectiveness of early NB skin-to-skin contact in the Kangaroo unit compared to conventional care in the neonatal unit, in relation to the growth and breastfeeding of very low weight NBs at 40 weeks corrected age. , Variables studied: mean weight gain (g/kg/day) from randomization to corrected term age.	At full-term GA there were no differences between groups regarding mean weight gain (g/kg/day) after randomization and breastfeeding rate. , Skin-to-skin contact in the Kangaroo unit is as effective as conventional care at the neonatal unit with no increase in mortality or morbidity in very low weight NBs.
Sharma et al.^(86)^	Randomized controlled clinical trial	n = 141 GA: ≤ 32 weeks Weight: < 1,000g Clinically stable	Two groups: 71 KWC Group and 70 to Intermediate Intensive Care Group	Compare growth and cost-effectiveness of skin-to-skin contact with intermediate intensive care	Average weight gain, as well as weight, length and head circumference at term corrected age were comparable in both groups, Initiating early shifting to Kangaroo ward is cost effective intervention and have huge monetary implication in resource poor countries

GA - gestational age; BW - birth weight; HR - heart rate; SpO_2 _- oxygen saturation; FiO_2 _- fraction of inspired oxygen; NB - newborn; CPAP - continuous positive airway pressure; TcPCO_2 _- transcutaneous carbon dioxide pressure; SSC -  skin-to-skin care ; NICU - neonatal intensive care unit; PTNB - preterm newborn; ETT - endotracheal tube; HFNC - high flow nasal cannula; rcO_2_ : Regional cerebral oxygenation; HR -  heart rate; cFTOE - cerebral fractional tissue oxygen extraction;  AT -axillary temperature; RR - respiratory rate; FTNB - full-term newborn; KC - kangoroo care;; PIPP - Premature Infant Pain Profile; KWC - kangorro ward care.

**Table 8S:** Studies included for multisensory stimulation recommendations

Author	Type of study	Sample	Interventions	Main outcomes	Main results
Kanagabasai et al.^(88)^	Randomized controlled	n = 50 GA: 28 - 36 weeks Weight: 1,000 to 2,000g	Two groups: Control Group (n = 25); Multisensory Stimulation Group (n = 25) submitted to interventions in five 12-minute sessions per week until hospital discharge	Neuromotor development evaluation	The multisensory intervention group had better muscle tone
Medoff-Cooper et al.^(89)^	Randomized controlled	n = 183 GA: 29 - 34 weeks Weight: not disclosure	Two groups: Control Group (n = 93); Multisensory Stimulation (ATVV) Group (n =90) to evaluate sucking organization PTNBs following a multisensory stimulation, ATVV = 10' of auditory (female voice), tactile (moderate touch stroking or massage) and visual (eye to eye) stimulation, followed by 5' of vestibular stimulation (horizontal rocking)	Infant sucking was digitally recorded	ATVV infants exhibited improved sucking organization during hospitalization
White-Traut et al.^(90)^	Randomized controlled	n = 195 GA = 29 to 34 weeks Weight: not disclosure	Two groups: Control Group (n = 95) H-Hope Group (n = 90): ATVV protocol performed twice more maternal participatory guidance sessions by a nurse-community advocate team daily for 6 consecutive weeks	Behavioral state (frequency of oral behaviors and time of alertness)	The intervention group showed greater frequency of oral behaviors and increased alertness
White-Traut et al.^(91)^	Randomized controlled	n = 198 GA: 29 to 34 weeks Weight: not disclosure	Two groups: Control Group (n = 102) H-Hope Group (n = 96)	Improved mother-baby interaction during feeding and play at 6-weeks corrected age	The intervention group had better mother-baby interaction
White-Traut et al.^(92)^	Randomized controlled	n = 182 GA: 29 - 34 weeks Average weight = 1,865g	Two groups: Control group (N = 94). H-Hope Group (n = 88): ATVV protocol performed twice daily for 6 consecutive weeks	Weight-length growth measured by weight and height	The intervention group had faster weight-length gain than the control group

GA- gestational age; ATVV- protocol Auditory, Tactile, Visual and Vestibular stimulus; PTNBs- preterm newborns; H-HOPE - Hospital to Home: Optimizing the Infant's Environment.

**Table 9S:** Studies included for multisensory skin-to-skin stimulation recommendations

Author	Type of study	Sample	Interventions	Main outcomes	Main results
Erdem et al.^(95)^	Randomized controlled	n = 28 GA: 26 - 32 weeks Weight: ≤ 1,000g	Two groups: Control Group (n = 14) Daily Mobilization Group (n = 14): Moyer-Mileur* protocol for 4 weeks (5 weekly sessions once a day)	Bone mineralization and anthropometric indices	The intervention group showed weight (p = 0.002) and height (p = 0.015) increase, as well as improved bone mineralization (p ≤ 0.001) compared to the control group
Tosun et al.^(96)^	Randomized controlled	n = 40 GA: 26 - 32 weeks Birth weight: 800 - 1,600g Low-risk PTNBs	Two groups: Control Group (n = 20) Intervention Group (n = 20): Moyer-Mileur protocol for 4 weeks (5 weekly sessions once a day)	Bone mineralization and anthropometric indices	The intervention group improved its bone mineralization (p ≤ 0.001) and anthropometric indices (p ≤ 0.001) compared to the control group
Vignochi et al.^(97)^	Randomized controlled	n = 30 GA: ≤ 35 weeks Weight: not disclosed	Two groups: Control Group (n = 15); Physiotherapy Group (n = 15): passive flexionextension movements associated with mild joint compression/decompression for 15 minutes 5 times a week (until NB reaches 2,000g) Bone metabolism: imbalance between bone formation and resorption was lower in the intervention group	Bone metabolism	Imbalance between bone formation and resorption was lower in the intervention group
Litmanovitz et al.^(98)^	Randomized controlled	n = 34 PTNBs GA: 28.6 ± 1.1 weeks Weight: 1,217g ± 55g	3 Groups: Group 1 Control (n = 10): no interventions Group 2 (n = 13): mobilizations twice a day Group 3 (n = 11): mobilizations once a day. Mobilization started at the NB's 08 ± 2.4 days of life and continued for 4 consecutive weeks Mobilization protocols: passive mobilization consisting of flexion-extension of the extremities (upper and lower limbs)	Bone mineralization assessed by ultrasound	There was decreased bone loss in Group 2 compared to the other groups (p = 0.03), suggesting that passive mobilization performed twice a day may prevent demineralization
Chen et al.^(99)^	Randomized controlled clinical trial	n = 16 GA: not disclosed Birth weight: ≤ 1,500g	2 Groups Control Group (n = 8): routine care Intervention Group (n = 8): submitted to the Moyer-Mileur* protocol.	Bone mineralization assessed by ultrasound	There was an increase in bone mineralization in the intervention group compared to the control group

GA- gestational age; PTNBs- preterm newborns; NB - newborn.

* Moyer-Mileur* protocol.is: Extension and flexion in the range of motion exercise against extremity resistance, performed for wrists, elbows, shoulders, ankles, knees, and hip-joints (acetabulofemoral joints), 5 days/week for 4 weeks with one session a day. Each activity repeated 5 - 8 times.
